# Risk Factors for Duty-Related Posttraumatic Stress Disorder among Police Officers in the Mt. Ontake Eruption Disaster-Support Task Force

**DOI:** 10.3390/ijerph17093134

**Published:** 2020-04-30

**Authors:** Tomoko Kamijo, Teruomi Tsukahara, Akihito Shimazu, Tetsuo Nomiyama

**Affiliations:** 1 Department of Preventive Medicine and Public Health, School of Medicine, Shinshu University, 3-1-1 Asahi, Matsumoto-shi, Nagano 390-8621, Japan; 2 Department of Occupational Medicine, School of Medicine, Shinshu University, 3-1-1 Asahi, Matsumoto-shi, Nagano 390-8621, Japan; tsukat@shinshu-u.ac.jp; 3 Faculty of Policy Management, Keio University, 5322 Endo, Fujisawa-shi, Kanagawa 252-0882, Japan; ashimazu-tky@umin.ac.jp

**Keywords:** posttraumatic stress disorder (PTSD), PTSD symptoms among police officers, peritraumatic situation, volcanic disaster

## Abstract

Mount Ontake in Nagano Prefecture, Japan erupted on 27 September 2014. Many police officers were called in for duty as a disaster-support task force. We investigated the association between the peritraumatic situation and posttraumatic stress disorder (PTSD) symptoms in these police officers. In January 2015, a health survey (OHS) on disaster stress related to the Mt. Ontake eruption disaster support work was distributed to all of the police officers and staff involved in the disaster support. We analyzed the 213 participants who had PTSD symptoms following the eruption and no missing OHS data. Logistic regression analyses were conducted to clarify the relationship between the participants’ symptom severity and their peritraumatic situation (i.e., stressors and daily support prior to the eruption, disaster-support work duties, and postdisaster stress relief). The symptom severity was associated with ‘more than seven cumulative days at work’ (odds ratio [OR] = 2.47, 1.21–5.06), ‘selecting drinking and/or smoking as stress relief after disaster-support work’ (OR = 2.35, 1.09–5.04), and ‘female’ (OR = 3.58, 1.19–10.77). As disaster-support work, ‘supporting the victims’ families’ (OR = 1.99, 0.95–4.21) tended to be associated with symptom severity. The number of days of disaster-support work, stress-relief behavior, and gender were associated with the severity of PTSD symptoms.

## 1. Introduction

The volcano of Mount Ontake (3067 m) in Nagano Prefecture, Japan erupted on 27 September 2014. Fifty-eight people died, five went missing, and many people were injured while climbing. The Japan Self-Defense Forces, the local police, and the fire departments were called in as a disaster-support task force. The search activities were extremely difficult due to the snow level, the mud of ashes, and also the risk of secondary disaster(s). Although only a few members of the disaster-support task force had special mountain equipment or rescue skills for high-altitude sites, none of the members of the task force died or was hurt by the mountain’s volcanic gas, but some developed altitude sickness and/or hypothermia [1].

In general, in addition to the survivors of a disaster, rescue workers such as police officers, firefighters, self-defense personnel, and emergency medical personnel are at increased risk of critical incident-induced stress because of their disaster-relief duties. In the report after Hurricane Katrina occurred in the U.S. in 2005, 26% of the police involved reported symptoms consistent with depression, and 19% reported symptoms of posttraumatic stress disorder (PTSD) [2]. After an airline crash, 13.5% of the medical personnel sent to assist the trauma victims had developed PTSD within 18 months [3]. Similarly, within 13 months of the 11 September 2001 terrorist attack in New York City, 21.7% of the many workers who were exposed to the attack or its aftermath developed depression and 16.7% developed PTSD [4].

PTSD is just one of the severe problems that rescue workers may face. Compared to the general population, the prevalence rate of PTSD among rescue workers is clearly higher [4,5,6], and PTSD is associated with not only higher psychiatric comorbidity but also physical illnesses [7]. The prevalence rate and severity of PTSD were observed to be associated with the type of traumatic event [8,9,10], general stress [11,12], stress-relief behavior (e.g., negative coping [13], addiction to alcohol [8,14,15,16], and smoking [17]), and resilience [18,19]. In recent years, the mental health of rescue workers who faced natural disasters and man-made mass violence has drawn increasing attention [2,4,5,6,20,21]. Protecting the physical, mental and emotional health of rescue workers is an important aspect of disaster recovery and of preserving the continuity of critical community functions [21]. To improve the quality of the mental-health care for disaster-recovery workers, more descriptive epidemiological studies of PTSD are necessary.

We conducted the present study to clarify the incidence and the severity of PTSD symptoms and to determine the relationship between the severity of PTSD symptoms and the peritraumatic situation among police workers who were called in for rescue and disaster-recovery duties after the Mt. Ontake eruption.

## 2. Participants and Methods

### 2.1. Setting and Participants

**Figure 1:**
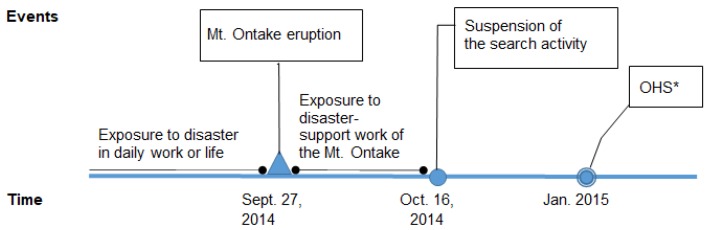
Exposure to the disaster and the time of investigation. * OHS: Health survey on disaster stress related to the Mt. Ontake eruption disaster-support work.

**Figure 2:**
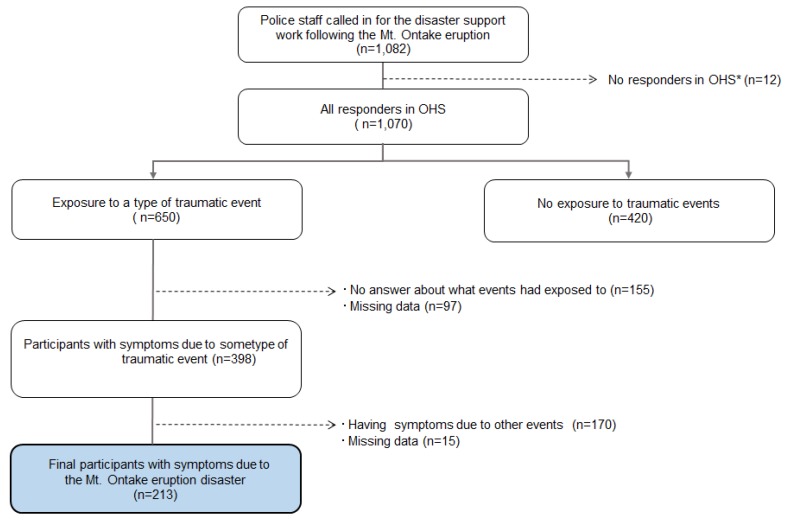
Flowchart of the process of identifying and selecting participants.

The search activities for the injured and deceased after the 27 September volcanic eruption were suspended on 16 October 2014 due to the worsened weather conditions (the snow level) and the increased risk of secondary disasters. At that time, there were 56 confirmed deaths and seven people still missing. Although the search activities were suspended, the support system for the families of missing persons continued. Three months after the suspension, in January 2015, a health survey (OHS) on disaster stress related to Mt. Ontake eruption disaster support work, which was an unsigned and self-recorded investigation, was distributed at the local police department (Figure 1). The police officers who had engaged in disaster-support work for more than one day were investigated. In the survey, 1070 of the 1082 participants were found to eligible for our analyses. In the OHS, 650 participants had exposure to some type of traumatic event (i.e., a natural disaster, including the Mt. Ontake eruption, vehicular accidents, assaults, or other critical events), and 398 of these 650 participants completed questionnaires regarding their current PTSD symptoms. We analyzed the 213 of these 398 participants who had PTSD symptoms due to their work on the Mt. Ontake eruption disaster-support task force (Figure 2). When we collected the data for analysis, there was no significant difference in the distribution of sociodemographic factors by exposure or to a specific type of events save for the participants’ work experience factor. Among the participants with PTSD symptoms, there was no significant difference in the sociodemographic factors by causal events (Mt. Ontake eruption/others).

### 2.2. Measures

#### 2.2.1. Traumatic Events and PTSD Symptom Severity

The posttraumatic-stress diagnostic scale (PDS) [22] provides a reliable score to measure the severity of PTSD for use in both clinical and research settings. Test items conform to the Diagnostic and Statistical Manual of Mental Disorders-Fourth edition (DSM-IV) criteria for PTSD. The diagnosis of PTSD is met when the PDS criteria A to F are all positive. The PDS assesses a subject’s PTSD symptoms in the past month [23]. We used the Japanese version of the PDS, the validity of which has been confirmed [24].

The items in the PDS first identify any causal traumatic events experiences for the respondent’s symptoms from among 11 extremely stressful events [23]. In the present study, we divided the traumatic events into three types: natural disasters, accidents, and other events (assaults, life-threatening illness, and others). The respondents’ current PTSD symptoms included exposures to traumatic events or stress both before and after the eruption (Figure 1). To investigate the PTSD symptoms due to the Mt. Ontake disaster, we had to separate the respondents’ current symptoms from their pre-existing symptoms that were due to past experiences, including the Kobe Earthquake in 1995, the Niigata Chuetsu Earthquake in 2004, and the Tohoku Earthquake and Tsunami in 2011.

A total PTSD symptom-severity score was generated by summing the individual respondent’s responses to the 17 symptom items, with the additional use of a four-point scale to assess re-experiment (criterion B), avoidance/paralysis (criterion C), and arousal enhancement (criterion D). The cut-offs for the symptom-severity rating were as follows. No rating: 0, mild: 1–10, moderate: 11–20, moderate to severe: 21–35, and severe: >36 [23]. In addition to a report of no symptoms, the score of 0 was given when a respondent reported experiencing a symptom only one time over the month-long period.

#### 2.2.2. The Peritraumatic Situations of the Participants with PTSD Symptoms from the Mt. Ontake Eruption Disaster

We assessed the peritraumatic situation of each of the participants with PTSD symptoms due to the Mt. Ontake eruption. The assessment included queries of the participant’s awareness of stress, the presence/absence of a personal support system for the participant in his or her daily life and in the workplace prior to the eruption, and the cumulative number of days that the participant engaged in the Ontake disaster. There were 15 types of duties as part of the recovery task force: (1) searching for missing people; (2) transportation; (3) inquest/corpse inspection; (4) responding to inquiries/information gathering on missing people; (5) support of the victims’ families; (6) management of victim’s personal belongings; (7) aviation unit; (8) setting up the communication system, (9) equipment; (10) traffic control; (11) general lodging and supplies for each unit; (12) supporting the local police station; (13) public relations; (14) supporting the medical personnel; and (15) command and general affairs (security headquarters, local headquarters). The participants sometimes did several tasks during the period.

The participants in this study were involved with duties (1–3) listed above, i.e., ‘search-and-rescue/transportation/corpse inspection’ as a direct traumatic stress and duties (5, 6), i.e., ‘Support of victim families/victim’s personal effects’ as secondary traumatic stress [25]. We also included the participants’ stress-relief behavior after the Mt. Ontake disaster as part of the peritraumatic situations. The following categories of stress-relief behavior were investigated by the OHS: (a) conversation(s) with a colleague who had experienced similar situations, (b) conversation(s) with a colleague without a similar experience, (c) conversation(s) with family members, (d) conversation(s) with friends, (e) exercise and hobbies, (f) rest, (g) alcohol and cigarettes. We focused on the participants’ conversations with other people and their use of alcohol and/or smoking; only a few people described engaging in exercise/hobbies or rest as a stress-relief behavior.

#### 2.2.3. Resilience

Resilience is considered a factor that helps prevent PTSD [18,19,26,27]. We measured our participants’ resilience by using the Connor–Davidson resilience scale (CD-RISC) (Japanese version), which comprises 25 items with confirmed reliability and validity [26,27].

### 2.3. Sociodemographic Factors

After the verification of multiple collinearities in a multiple logistic regression analysis conducted to estimate their contributions to the severity of PTSD symptoms, we used the participants’ gender, living situation, and years of work experience as a police officer or staff as independent factors.

### 2.4. Statistical Analyses

We used logistic regression to identify factors that are associated with the severity of PTSD symptoms due to the participants’ Mt. Ontake eruption disaster-support work. We performed single and multiple logistic regression analyses for the 213 participants who had PTSD symptoms (PDS score ≥1) due to the Mt. Ontake eruption disaster. In the logistic regression analyses, the independent factors were peritraumatic situations, resilience, and sociodemographic factors. Resilience was divided into high, middle, and low groups based on the participants’ CD-RISC total score, and the high group was used as the reference. The SPSS Statistics 25 program was used for all statistical analyses.

### 2.5. Ethical Approval

This study and protocol were approved by the Ethics Review Committee of Shinshu University School of Medicine (No. 3963), and the study conformed to the tenets of the Declaration of Helsinki.

## 3. Results

### 3.1. The Participants’ Characteristics

Table 1 summarizes the characteristics of the 213 participants with PTSD. Males accounted for 91.1% of the participants. The most common age group was the 30 s; 60.1% of the participants were unmarried, 45.1% lived alone, 84.0% were police officers, and the years of work experience was <10 years in 48.4% of the participants. In PTSD symptom-severity rating, ‘no rating’ was the score for 73.2% of the participants, ‘mild’ was achieved by 25.8%, and ‘moderate’ by 0.9%. None of the participants had severe PTSD symptoms due to the Mt. Ontake eruption.

### 3.2. The Relationship between PTSD Symptoms and the Participants’ Peritraumatic Situations

Table 2 lists the factors associated with the PTSD symptoms along with the peritraumatic situation factors. In the crude model, gender, work stress before the disaster, cumulative days on duty, and the duty of supporting the victims’ families each had a significant impact on the symptom severity. In the fully adjusted model, the following three factors had a significant effect on the severity of the participants’ symptoms: Female gender (odds ratio [OR] = 3.58, 95% confidence interval [CI]: 1.19–10.77), cumulative days on duty ≥7 (OR = 2.47, 95%CI: 1.21–5.06), and drinking or smoking as stress relief (OR = 2.35, 95%CI: 1.09–5.04). Pre-existing work stress was not associated with the symptom severity in the adjusted model. Resilience contributed to neither model.

## 4. Discussion

We were able to identify (1) the severity of the participants’ PTSD by using the PTSD symptom score (the PDS), and (2) factors associated with the severity of the PTSD symptoms due to the Mt. Ontake disaster. The factors associated in the adjusted model were gender, cumulative days on the job performing disaster-support work, and drinking and smoking as stress-relief behaviors.

### 4.1. The Severity of the PTSD Indicated by the PDS

The lifetime prevalence of PTSD among community residents is 6.8% in the U.S. [28] and 1.3% in Japan [29]. Our present analyses of police officers and staff revealed that none of the participants experienced moderate or severe PTSD symptoms, and none were diagnosed as having PTSD. In addition, the mean PDS score among the participants was extremely low compared to the scores of aid workers in developing countries [30] and a non-PTSD group of subjects with mental illness [30,31]. In general, the risk of developing PTSD due to natural disasters has been considerably lower than other traumatic events such as accidents and assault (physical, sexual) in both the US National Comorbidity Survey [8] and a Japanese survey [29]. This result was also confirmed by the responses on the OHS by our participants.

Another reason for the low incidence of PTSD symptoms among the present participants is that their prior emergency training including their daily police duties and seminars may contribute to a low risk of PTSD symptoms. Fullerton et al. [32] described the efficacy of sufficient training before missions. Several studies have reported that the prevalence of PTSD after disasters is lower among rescue workers than in the general public [33,34]. In a survey of 9/11 first responders, nontraditional responders who had not been trained (e.g., construction workers) were twice as likely to develop PTSD compared to police [35]. It has been reported that support staff (fire staff [20,36,37] and disaster-dispatch medical teams [38]) dispatched to an afflicted area also did not show a high risk of PTSD symptoms.

We also consider the low incidence of PTSD symptoms among police and the low risk of natural disasters. Even for a trained responder, natural disasters could cause PTSD. The type and level of exposure to critical events vary among individual experiences. It has also been reported that first responders who have experienced a major disaster (e.g., natural disaster, traffic accident, terrorist attack) have a higher lifetime prevalence of PTSD than first responders who have not experienced a major disaster or the general population [4,5]. Evidence shows that, when encountering the same disaster, police officers have a lower tendency to PTSD than the general population, yet a higher lifetime risk.

### 4.2. The Relationship between PTSD Symptoms Due to the Mt. Ontake Eruption Disaster and the Participants’ Peritraumatic Disaster-Support Work

We examined the associations between PTSD symptoms (PDS score greater than one: mild or more symptoms in the PDS symptom-severity rating) and the peritraumatic situation of the participants’ specific disaster-support work. The results of the adjusted model indicated that female gender was most relevant to the mild symptoms. This risk among female disaster staff is consistent with past reports [8,39,40,41], but in the present study this result might have been influenced by the low number of females among the participants. The wide range of 95% confidence intervals of female gender also suggests that there are individual differences. There may be confounders that are not fully adjusted.

A large number of cumulative days of disaster-support work was the most relevant of the variables of the peritraumatic situation. Our findings suggest that government entities can help prevent PTSD among their disaster personnel by managing the length of the on-duty periods. Examples of the steps that municipal and prefectural governments can take include limiting the duration of the dispatch period for first responders; personnel could receive periodic care during their disaster-support work; and efforts should be made to place the right person in the right post in consideration of gender differences.

Despite the danger to their own lives and the difficult contact with corpses and human remains, the participants in this study were on duty for days while they were searching and transporting survivors/bodies and performing autopsy work. These activities did not contribute significantly to having mild or worse symptoms of PTSD. In fact, the participants who engaged in searches, transport, and autopsies showed higher resilience scores (CD-RISC) than the participants who engaged in other tasks, as well as low psychological distress scores (K6, GHQ-12) on the OHS. We speculate that relatively mentally healthy staff were in charge of the disaster-recovery work after the Mt. Ontake eruption. These tasks require special skills, and we thus also speculate that these police staff had disaster preparedness from daily training. Another study indicated that non-traditional responders were twice as likely to develop PTSD compared to police dealing with the same disaster [35].

Being on duty supporting the victims’ families may contribute to mild or worse PTSD symptoms; this might be the influence of secondary traumatic stress [25]. Secondary traumatic stress is an increasingly important point of research related to health effects among first responders [42]. The risk of PTSD symptoms might be high because the period on duty for supporting victims’ families is longer than the period for other work, and the psychological toll can be high. In a survey of all of the firefighters in Hyogo Prefecture, Japan after the Kobe earthquake, there were considerable effects of psychological distress evoked by citizens’ accusations and complaints, in addition to the stress from the damage to the firefighters’ own homes from the disaster [43]. A report from the police in New Zealand indicates that distress at survivor reactions predicted less helpful survivor support and, was equal with peritraumatic distress, was the strongest secondary traumatic stress predictor [44]. The secondary traumatic stress was linked to dispositional empathy, especially the self-oriented emotional disposition of empathy [45]. Even in the present investigation, 53.1% of the participants reported that they felt “helplessness” from both the traumatic event and the influence of subjective distress. Police officers have many opportunities to interview victims of crimes and disasters. We are concerned about the risk of secondary traumatic stress due to compassion fatigue from supporting victims [25,46,47], and we propose that methods for preventing PTSD are necessary for not only direct exposure to a disaster but also for the effects of indirect trauma exposure.

Herein, choosing drinking and smoking for stress relief after the disaster-support work was significantly related to mild or worse PTSD symptoms, after a large number of cumulative workdays. Kessler et al. reported that PTSD tended to coexist with alcohol dependence; 52% of their male subjects with PTSD had comorbid symptoms of alcohol dependence [8]. Relationships were confirmed between PTSD in police officers and an increase in alcohol intake [14] and a reduction in the possibility of smoking cessation [17]. In Japan, increases in alcohol intake, alcohol dependency, and smoking volume were reported in affected people after disasters [15,16]. Regarding the increase in alcohol intake due to disaster exposure, it is said that drinking behaviors are used as a method of coping against the influence of traumatic events [48]. The association between traumatic stress and drinking/smoking has been confirmed in prior studies. As depression is also associated with alcohol consumption, further investigation is needed to take into account pre-existing mental illness too. This study did not confirm the amount of alcohol intake or smoking, but it did provide a trend in selection. It is therefore necessary to pay attention to changes in the drinking behavior of personnel in police organizations after their exposure to critical incidents, although these individuals might be still in the acute-stress response stage (which may disappear with time). It may also be effective to provide education to police about stress-relief methods as substitutes for drinking and smoking in advance of disasters.

### 4.3. Strengths and Limitations

Our present findings could be used to help police officers dispatched to disasters from a single organization with a high response rate. Our search of the PubMed database revealed no study of PTSD caused by a volcanic disaster among police officers. We were able investigate PTSD that was due specifically to the participants’ Mt. Ontake eruption disaster-support work, excluding influences from other events. A relationship between PTSD symptoms and peritraumatic situations was revealed after the adjustment for factors including those to consider before, during, and after exposure to a traumatic event.

However, there are some study limitations to consider. (1) Because this was only a cross-sectional study and an a posteriori investigation, a causal relationship between the subjects’ peritraumatic situations and PTSD symptoms cannot be confirmed. (2) Regarding the severity of PTSD symptoms, this analysis used a cut-off value that is synonymous with one or more symptoms, because the number of serious cases was extremely small. (3) We did not determine the exact number of days that had elapsed from the occurrence of each participant’s personal disaster experience to the investigation day. Thus, the number of days that had elapsed was not taken into consideration in the assessment of the association with PTSD symptom severity. (4) We have not been able to confirm the respondent’s medical history such as the original mental illness. (5) There were 12 blank responses in the OHS. Because the OHS is an organizational survey, it cannot be denied that such a collection method could have affected the participants’ answers, although the participants were informed of the protection of their personal information. In the OHS, some of the staff members did not name the causal traumatic event even though they answered questions about the severity of PTSD symptoms. Some staff answered that they experienced “no trauma” in the descriptive question about details of the traumatic experience. There may be a sense of resistance to answering questionnaires because of PTSD symptoms, including re-experiencing. Consideration of these factors is necessary when using group surveys about PTSD symptoms. (6) The participants’ PDS score was low. It is not without the possibility that the individual’s feelings of pain were discounted in the hope that this would reflect well on their roles as a job as a police officer. (7) The number of participants who were eligible for the analyses was somewhat low, but the participants reflected the entire spectrum on the distribution of sociodemographic factors of the OHS. (8) In this study, we couldn’t divide the task of search and rescue, transportation, or autopsy for two reasons, though there are differences in the dangers of work in question. First, many participants were engaged in multiple tasks with large ranges of work intensity and risk during the search activity. Second, we avoid an excessive number of factors for the quality of the analysis model.

## 5. Conclusions

None of the participants reported having severe symptoms of PTSD due to the Mt. Ontake eruption. Our analyses demonstrated that several factors contributed to the PTSD symptoms from peritraumatic situations. The most relevant was female gender, followed by a large number of cumulative workdays and drinking or smoking as stress-relief behaviors. Aspects of the specific disaster-support duties and the participants’ daily work stress were also related. Our findings may be useful for the prevention, for example, managing the length of the on-duty period, and early detection of PTSD among disaster workers.

## Figures and Tables

**Table 1:** Characteristics of the participants (n = 213).

Variable	Category		n	(%)
*Sociodemographic factors*				
Sex				
	Male		194	(91.1)
	Female		19	(8.9)
Age				
	20–29		63	(29.6)
	30–39		87	(40.8)
	40–49		45	(21.1)
	50+		18	(8.5)
Marital status:				
	Married		83	(39.0)
	Unmarried		128	(60.1)
	Bereavement or divorced		2	(0.9)
	No answer		0	(0.0)
Living situation:				
	Living alone		96	(45.1)
	Living with someone		117	(54.9)
Job title:				
	Police officer		179	(84.0)
	Office staff		27	(12.7)
	No answer		7	(3.3)
Years of work experience:				
	1–9		103	(48.4)
	10–19		72	(33.8)
	20+		38	(17.8)
*PTSD symptom severity rating:*				
	No rating, 0		156	(73.2)
	Mild, 1–10		55	(25.8)
	Moderate, 11–20		2	(0.9)
	Moderate to severe, 21–35		0	(0.0)
	Severe, 36+		0	(0.0)
*Peritraumatic situations*				
Disaster support duties:				
	Search and rescue or transportation or autopsy	None	100	(46.9)
		Yes	113	(53.1)
	Support for the victim’s family or the remains of victims	None	139	(65.3)
		Yes	74	(34.7)
	Cumulative days on the work	<7 days	90	(42.3)
		≥7 days	123	(57.7)
Stressors and supports before the disaster:				
	Work stress	None	170	(79.8)
		Have	43	(20.2)
	Family or personal stress	None	161	(75.6)
		Have	52	(24.4)
	Supporter for usual work	None	37	(17.4)
		Yes	176	(82.6)
	Supporter for family life or personal problems	None	46	(21.6)
		Yes	167	(78.4)
Stress relief behavior after the disaster:				
	Conversation with family or colleagues	None	64	(30.0)
		Yes	149	(70.0)
	Drinking or smoking	None	164	(77.0)
		Yes	49	(23.0)
*Resilience*				
CD-RISC score:				
	High, 62+		68	(31.9)
	Medium, 50–61		83	(39.0)
	Low, <50		62	(29.1)
CD-RISC: Connor–Davidson Resilience Scale				

**Table 2:** The relationship between a posttraumatic-stress diagnostic scale (PDS) score greater than one and the peritraumatic situation of disaster support work among participants with symptoms after the Mt. Ontake eruption disaster.

Variable	Category		Crude Model				Adjusted Model			
		n	OR	(95%CI)	*p*-Value	*p* for Trend	OR	(95%CI)	*p*-Value	*p* for Trend
*Sociodemographic factors*										
Sex										
	Male	194	1.00				1.00			
	Female	19	3.48	(1.33–9.06)	0.01		3.58	(1.19–10.77)	0.02	
Living situation										
	Live alone	96	1.00				1.00			
	Live with someone	117	0.80	(0.44–1.47)	0.44		0.79	(0.38–1.65)	0.53	
Years of work experience										
	1–9	103	1.00				1.00			
	10–19	72	0.96	(0.49–1.90)	0.96	0.90	0.91	(0.40–2.09)	0.82	0.89
	20+	38	0.96	(0.41–2.22)	0.96		0.94	(0.35–2.55)	0.90	
*Peritraumatic situations*										
Disaster support duties:										
Search and rescue, transportation or autopsy	None	100	1.00				1.00			
	Yes	113	0.89	(0.48–1.63)	0.70		1.35	(0.61–2.99)	0.46	
Support for the victim’s family or the remains of victims	None	139	1.00				1.00			
	Yes	74	2.09	(1.12–3.89)	0.02		1.99	(0.95–4.21)	0.07	
Cumulative days at work	<7 days	90	1.00				1.00			
	≥7 days	123	2.31	(1.20–4.46)	0.01		2.47	(1.21–5.06)	0.01	
Stressor and supports before the disaster:										
Work stress	None	170	1.00				1.00			
	Have	43	2.12	(1.05–4.31)	0.04		1.58	(0.66–3.79)	0.30	
Family or personal stress	None	161	1.00				1.00			
	Have	52	1.01	(0.50–2.05)	0.98		0.76	(0.33–1.76)	0.52	
Supporter for usual work	None	37	1.00				1.00			
	Yes	176	0.53	(0.25–1.12)	0.97		0.51	(0.18–1.40)	0.19	
Supporter for family life or personal problems	None	46	1.00				1.00			
	Yes	167	0.61	(0.30–1.23)	0.12		1.02	(0.39–2.65)	0.97	
Stress relief behavior after the disaster:										
Conversation with family or colleagues	None	64	1.00				1.00			
	Yes	149	0.73	(0.38–1.39)	0.33		0.85	(0.40–1.80)	0.66	
Drinking or smoking	None	164	1.00				1.00			
	Yes	49	1.65	(0.83–3.28)	0.16		2.35	(1.09–5.04)	0.03	
*Resilience*										
CD-RISC score	High, 62+	68	1.00				1.00			
	Medium, 50–61	83	0.96	(0.45–2.06)	0.93	0.13	0.98	(0.43–2.25)	0.96	0.44
	Low, <50	62	1.79	(0.83–3.84)	0.14		1.42	(0.59–3.42)	0.43	
